# When we shouldn’t borrow information from an existing network of trials for planning a new trial

**DOI:** 10.3389/fphar.2023.1157708

**Published:** 2023-04-27

**Authors:** Fangshu Ye, Chong Wang, Annette M. O’Connor

**Affiliations:** ^1^ Department of Statistics, Iowa State University, Ames, IA, United States; ^2^ Department of Veterinary Diagnostic and Production Animal Medicine, Iowa State University, Ames, IA, United States; ^3^ Department of Large Animal Clinical Sciences, Michigan State University, East Lansing, MI, United States

**Keywords:** network meta-analysis, clinical trial design, evidence synthesis, sequential analysis, bovine respiratory disease, antibiotic

## Abstract

**Introduction:** To achieve higher power or increased precision for a new trial, methods based on updating network meta-analysis (NMA) have been proposed by researchers. However, this approach could potentially lead to misinterpreted results and misstated conclusions. This work aims to investigate the potential inflation of type I error risk when a new trial is conducted only when, based on a *p*-value of the comparison in the existing network, a “promising” difference between two treatments is noticed.

**Methods:** We use simulations to evaluate the scenarios of interest. In particular, a new trial is to be conducted independently or depending on the results from previous NMA in various scenarios. Three analysis methods are applied to each simulation scenario: with the existing network, sequential analysis and without the existing network.

**Results:** For the scenario that the new trial will be conducted only when a promising finding (*p*-value <5% ) is indicated by the existing network, the type I error risk increased dramatically (38.5% in our example data) when analyzed with the existing network and sequential analysis. The type I error is controlled at 5% when analyzing the new trial without the existing network.

**Conclusion:** If the intention is to combine a trial result with an existing network of evidence, or if it is expected that the trial will eventually be included in a network meta-analysis, then the decision that a new trial is performed should not depend on a statistically “promising” finding indicated by the existing network.

## 1 Introduction

Network meta-analysis (NMA) is a tool increasingly used in human and animal health to understand the comparative effect of interventions. One of the unique features of network meta-analysis is the ability to generate estimates of comparative efficacy when no direct clinical trial exists. Another advantage of meta-analysis, not just network meta-analysis, is the ability to leverage evidence to increase the power of relative comparisons. Methods also exist to plan a randomized clinical trial specifically to update a pairwise meta-analysis (Sutton et al., 2007; Roloff et al., 2013) or network meta-analysis; such approaches require smaller sample sizes to achieve a certain power and are resource saving (Nikolakopoulou et al., 2014; Nikolakopoulou et al., 2016; Salanti et al., 2018).

An interesting question that arises from the latter use of meta-analysis is how should investigators leverage prior evidence and network meta-analysis appropriately to help investigators design a resource-saving trial? One concern with the approach is that investigators may misuse the idea of leveraging evidence from networks of trials in designing and analysing the new trial. We hypothesise that while it is a valid decision to use evidence from the existing network to increase the power of comparison, the decision to conduct a new trial with a particular comparison, should not be motivated by the prior results if the new trial is to be analyzed with the existing network. The distinction between these use cases when selecting the comparison for the new trial can be challenging for trialists and clinicians to recognize.

In particular, there is concern that researchers will conduct a trial based on observing promising indirect estimates obtained from network meta-analysis. The idea is that upon seeing a promising indirect estimate, the goal could be to obtain a direct estimate that would provide enough power to reach statistical significance (Whitemore et al., 2019). Such a scenario might be that the new trial is motivated by observing a *p*-value for the indirect relative effect that is <0.1 i.e., a promising indirect result. We hypothesise that such a use case will result in an inflated type I error risk in the hypothesis testing process for the comparison in the NMA. Further, we hypothesise that if the decision to conduct a trial of a particular comparison has been made independent of the existing network, it is then reasonable to use the existing network to increase the precision of the estimate. We also evaluate if trial sequential analysis, a method used in pairwise meta-analysis to control the maximal risk of type I error, could be an effective tool for use cases that do inflate the type 1 error risk (Nikolakopoulou et al., 2018).

Overall the objective of this study is to investigate the type I error risk inflation in different use scenarios with different analysis methods as approaches to controlling error risk inflation. With this information, trialists and clinicians can be mindful when using information from meta-analysis to design a new trial.

## 2 Materials and methods

### 2.1 Data description

**FIGURE 1:**
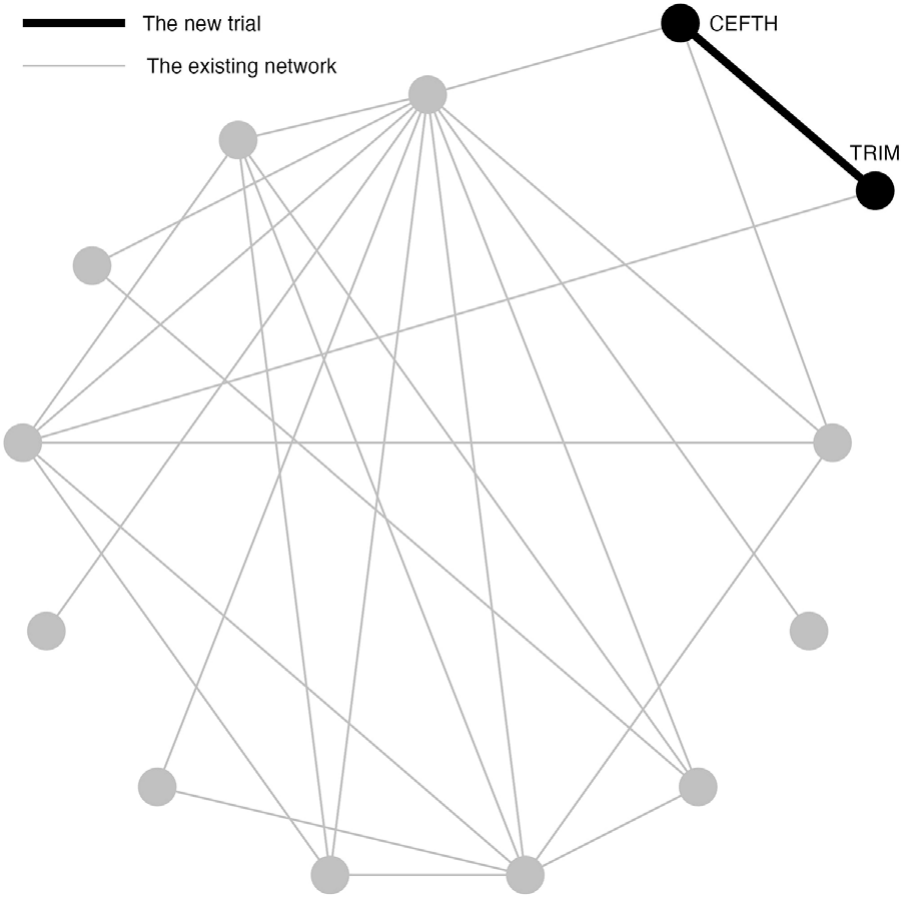
An existing network of interventions for the treatments of Bovine Respiratory Disease (BRD) in feedlot cattle with a new study between CEFTH(A) and TRIM(B) where A and B had no direct comparison in the existing network.

A previously published network of interventions for the treatment of Bovine Respiratory Disease (BRD) in feedlot cattle is used as an illustrative example for the problem of interest (O’Connor et al., 2016). The network comprises 98 trials and 13 treatments in total. Most trials contain two arms and eight trials contain three arms. The network plot is shown in Figure 1. Arm-level data are available and the outcome is a dichotomous health event. To compare treatments, the log odds ratios for pairwise comparisons are calculated.

We demonstrate the problem of inflated type I error risk using two simulation studies.

### 2.2 Simulation study 1: example scenario of NMA

Simulation Study 1 is designed to demonstrate a situation when a new two-arm trial is planned that contains two treatments in the existing network, but these two treatments are not directly compared in the network (Figure 1). In Simulation Study 1, the new two-arm trial contains Ceftiofur hydrochloride (CEFTH) and Trimethoprim (TRIM) as treatments. These treatments appeared in the existing BRD network but without existing direct comparison, therefore the estimated relative effect size is based only on indirect evidence. The estimate of the relative effect size of CEFTH and TRIM was obtained by the frequentist NMA and a *p*-value of 0.08 indicated a “promising” relative effect size that was not significant at 0.05 level. To simplify the notation, we replace CEFTH and TRIM with A and B.

Simulation Study 1 assesses the type I error risk when the decision to conduct a new trial either depends upon or, is independent of, a hypothesis test for a difference between two treatments in the existing NMA. Three scenarios are considered based on the hypothesis test result from the existing NMA: Scenario 1 is that the new trial will only be conducted when the *p*-value <0.1 , Scenario 2 is that the new trial is only conducted if the *p*-value is between 0.05 and 0.1, and Scenario 3 is that the decision to conduct the new trial is independent of the *p*-value of the observed relative effect. The rationale for Scenario 1 is that a new trial with direct comparison is motivated by a “promising” indirect comparison *p*-value that is < 0.1. The rationale for Scenario 2 is that there might be not enough motivation to do the new trial if the *p*-value is already below 0.05 for the comparison of interest. The hypothesis test that the *p*-value refers to is based on the existing NMA. The type I error risk for each scenario is assessed using three analysis methods: without the existing network (new trial only analysis); with the existing network (network meta-analysis), and sequential analysis with the existing network. The details of these three analysis methods are explained in the *Analysis method* section below.

### 2.3 Simulation procedure

The true log odds ratio between A and B is set to be 0 in order to assess the type I error risk. Four values of total sample sizes *n* (50, 100, 150, and 200) are considered for the new trial. The steps of the simulation procedure are described as below:

1. From a network meta-analysis of the existing network, the risk of the event in treatment *j* can be estimated and denoted as *p*_*j*_. The risk of the event for B is set to be the same as A, i.e., *p*_*A*_ = *p*_*B*_.
2. For each treatment group *j* and total sample size *n*_*s*,*j*_ in study *s* of our existing network, we replace the number of events *r*_*s*,*j*_ with a random number generated from Binom (*n*_*i*_, *p*_*j*_). These are our existing simulated data.
3. Using the simulated network data, we conduct the network meta-analysis and test the hypothesis about the indirect comparison of A and B. The null hypothesis (*H*_0_) is that the effect size is 0 between A and B.
4. If the *p*-value is <0.1 we extract the Z value as *z*_1_ for future use and proceed to the next step, which simulates a new trial and obtains a direct comparison of A to B. If the *p*-value is ≥0.1 the decision is made not to proceed, i.e., not to conduct a new trial and this round of simulation will be removed from the result calculation. This step simulates the idea that a decision is made to conduct the trial based on a promising *p*-value.
5. Generate a new trial to simulate *r*_*i*_ from Binom (*n*/2, *p*_*i*_), *i* ∈ {*A*, *B*} for treatment A and B respectively, and analyze the simulated new trial using logistic regression. This step represents analyzing the trial without the existing network. Use an indicator to denote if the direct comparison has a *p*-value of ≥0.05 = 0 or <0.05 = 1.
6. Add the data of the new trial from step 5 to the existing simulated network data to represent a row of study-level data, i.e., a new direct comparison. Use NMA to analyze the combined data. This step represents analyzing the trial with the existing network without an error adjustment method. Use an indicator to denote if the comparison of A to B from the NMA has a *p*-value of ≥0.05 = 0 or <0.05 = 1. Extract the Z value as *z*_2_ for future use.
7. For the fixed total sample size *n*, obtain the corresponding *c*_1_ and *c*_2_ from Table 1. Use an indicator to denote if (*z*_1_ > *c*_1_ or *z*_2_ > *c*_2_) = 1 else 0. This step represents for analysing the trial using the sequential analysis.
8. Repeat steps 1–7 for 100,000 times.
9. Calculate the proportion of each indicator equal to 1. The proportion of 1’s in step 5, 6 and 7 estimate the type I error risk for three analysis approaches: without the existing network (new trial only analysis); with the existing network (network meta-analysis), and sequential analysis with the existing network. This is referred to as Scenario 1.
10. To simulate Scenario 2: the new trial will be conducted when the *p*-value in step 3 is between 0.05 and 0.1, go through steps 1–9 but revise step 4, i.e., change the threshold for *p*-value to be between 0.05 and 0.1. This is referred to as Scenario 2.
11. To simulate Scenario 3: the conduct of a new trial does not depend on the results of the indirect comparison in the existing network, go through steps 1–9 but omit step 4, i.e., the decision to conduct the trial is independent of the *p*-value of the indirect comparison. This is referred as Scenario 3.

**TABLE 1:** Sequential boundary for different total sample sizes of new trial in Simulation Study 1.

Total sample sizes of new trial	c_1_	c_2_
50	2.10	2.05
100	2.24	2.03
150	2.37	2.01
200	2.49	1.99

### 2.4 Analysis methods

Three methods are applied to analyze the simulated trial data: without the existing network (new trial only analysis); with the existing network (network meta-analysis), and sequential analysis with the existing network. A logistic regression is applied to analyze the new trial only without the existing network. For NMA with existing network, the fixed-effect frequentist NMA is implemented by using the package “netmeta” (Balduzzi et al., 2023) in R. The sequential analysis with NMA is described below.

### 2.5 Sequential analysis

The TSA program (Copenhagen Trial Unit Centre for Clinical Intervention Research, Denmark) (Thorlund et al., 2021) is a popular approach to address multiple testing error control in pairwise meta-analysis. The program provides a monitoring boundary, which is the collation of boundaries for the Z-curve, to adjust the boundaries for the Z values for sequential tests, and therefore allowing the overall type I error risk to be controlled to the desired maximum risk. Take a pairwise meta-analysis with two trials, for example, let *Z*_1_ and *Z*_2_ represent for the Z values for two tests. We need to find two values for the boundary, *c*_1_ and *c*_2_ for which

$$\text{Pr}\left(|Z_{1}|\geq c_{1}\text{ or }|Z_{2}|\geq c_{2}\right)\leq \alpha$$

is satisfied under the *H*_0_. This is equivalent to finding two maximum type I error risks, *α*_1_ and *α*_2_, that sum to *α* and where

$$\text{Pr}\left(|Z_{1}|\geq c_{1}\right)\leq \alpha_{1}$$

$$\text{Pr}\left(|Z_{2}|\geq c_{2},|Z_{1}|\leq c_{1}\right)\leq \alpha_{2}$$

under the *H*_0_.

One simple way to calculate the *α*_1_ and *α*_2_ is the *α*-spending function. There are several options for the *α*-spending function. The function implemented by the TSA program is given by the expression

$$\alpha \left(\text{IF}\right)=2-2\Phi \left(Z_{1-\alpha /2}/\sqrt{\text{IF}}\right)$$

where 0 < IF ≤ 1, Φ is the standard normal cumulative distribution function and the information fraction (IF), which is calculated by dividing the accumulated information by the required information size. For our example, we use sample size as information size. The boundary calculated by this *α*-spending function has been first proposed by O’Brien and Fleming (O’Brien and Fleming, 1979) for equal increments of IF. Lan and DeMets (Gordon Lan and DeMets, 1983) later have revised the approach to allow for flexible increments in IF. Therefore, the monitoring boundaries produced by this *α* spending function are referred to as the Lan-DeMets monitoring boundaries or O’Brien-Fleming monitoring boundaries (Thorlund et al., 2017).

The TSA is an approach for pairwise meta-analysis only and does not apply to network meta-analysis. Therefore, we have modified the approach to be applicable to NMA with the same alpha-sparing approach. We use the term “sequential” or “sequential analysis” to refer to the modified methods for NMA.

In Simulation Study 1, A and B are the two treatments in the new trial. The total sample sizes of treatment A and B in the existing network are 180 and 233 respectively. To conduct the sequential analysis, one interim analysis and one final analysis are considered. Thus, the number of hypothesis tests in the sequential analysis is two: the first is conducted on the indirect estimate of A and B in the existing network, and the second is conducted after adding the new trial. The *H*_0_ is that the effect size is 0 between A and B for both tests. To split *α* into *α*_1_ and *α*_2_, we pre-define a reasonable required information size. Suppose the total sample size for the new trial is *n*, our assumption is that the total sample sizes after adding the new trial, 413 + *n*, has sufficient power to detect the assumed difference between A and B. With the required sample size and the accumulated sample sizes for each hypothesis test, the TSA program is capable of calculating *c*_*i*_, which is the boundary for the test statistics *Z*_*i*_ in i-th test (i ∈ {1, 2}). For *n* ∈ {50, 100, 150, 200}, please see the corresponding *c*_*i*_ in Table 1.

### 2.6 Simulation study 2: example scenario of pairwise meta-analysis

**FIGURE 2:**
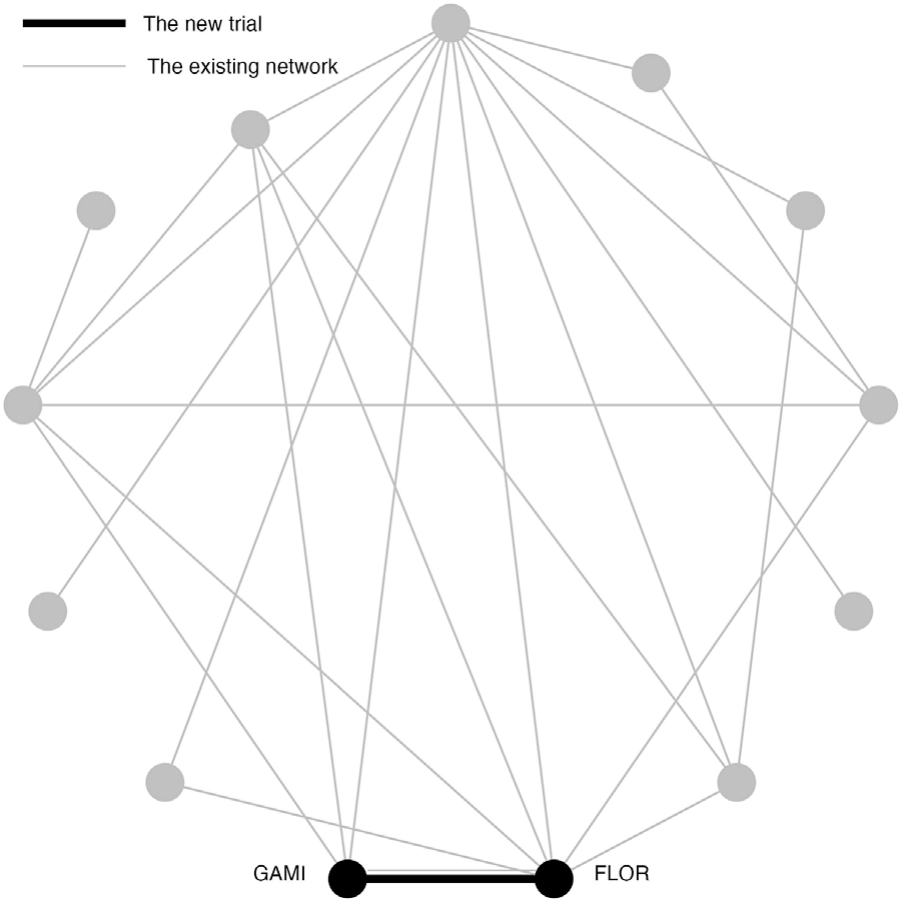
An existing network of interventions for the treatments of Bovine Respiratory Disease (BRD) in feedlot cattle with a new study between GAMI(C) and FLOR(D) where C and D had direct comparison in the existing network.

The purpose of Simulation Study 2 is to illustrate the inflation of type I error risk in a pairwise meta-analysis, where the original TSA method is applicable. In Simulation Study 2, the new two-arm trial contains Gamithromycin (GAMI) and Florfenicol (FLOR) (Figure 2). Simulation Study 2 differs from Simulation Study 1 because these treatments had a direct trial in the existing network, and a *p*-value of 0.096; a “promising” but not significant relative effect size. To simplify the notation, we replace GAMI and FLOR with C and D. Frequentist pairwise meta-analysis (PMA) of this comparison is applied in Simulation Study 2 because a direct comparison does exist.

In Simulation Study 2, the new trial compares C and D. The number of trials that directly compare C and D in the existing network is one and the sample size for that trial is 602. Single trial analysis of a two arm trial can be viewed as a special pairwise meta-analysis. Therefore, the number of hypothesis tests in TSA is 2: the first is conducted using the existing trial and the second is conducted combining the existing trial with the new trial. The *H*_0_ is that the effect size is 0 between C and D for both tests. To split *α* into *α*_1_ and *α*_2_, we need to determine the required information size. Assuming a 39% control event rate and a 10% risk reduction with 80% power and a 0.05 two sided *α*, the required information size is calculated to be 705 from the TSA program. To ensure that the accumulated sample size after adding the new trial equals the required information size, the total sample size for the new trial is set to be 103. We split a pre-defined maximum risk, 0.05, into 0.0339 for the first hypothesis test, and 0.0161 for the second hypothesis test. The boundary for the Z values is adjusted to 2.16 and 2.04 individually.

This simulation is designed for Simulation Study 2 of the new two-arm trial, which contains C and D as treatments. As before, there are three scenarios based on the rationale for conducting the new trial and the type I error risk is estimated under three analysis methods: new trial only estimate; meta-analysis with the existing data, and sequential analysis. However, this differs from Simulation study 1 as there is an existing estimate in the network and the last two analyses are pairwise meta-analysis.

As with Simulation Study 1, the log odds ratio between C and D is set to be 0 in the simulation. The risk of C is estimated to be 0.29 from the existing NMA. Therefore, the risk of D is set to be the same as C, i.e., *p* = *p*_*C*_ = *p*_*D*_ = 0.29 The simulation process is conducted as below:

1. In the existing trial, the sample size of treatment C and D is 297 and 305 individually. We replace the number of events in two groups with the random numbers generated from Binom (291, *p*) and Binom (305, *p*). This is the existing simulated trial.
2. We test the hypothesis about the comparison of C and D using the simulated trial. The null hypothesis (*H*_0_) is that the effect size is 0 between C and D.
3. If the *p*-value is <0.1 we extract the Z value as *z*_1_ for future use and proceed to the next step, which simulates a new trial and obtains a direct comparison of C to D. If the *p*-value is ≥0.1 the decision is made not to proceed, i.e., not to conduct a new trial and this round of simulation will be removed from the result calculation. This step simulates the idea that a decision is made to conduct the trial based on a promising *p*-value.
4. Generate a new trial to simulate the number of events from Binom (51, *p*) and Binom (52, *p*) for group C and D respectively, and analyze the simulated new trial using logistic regression. This steps represents for analyzing without the existing trials. Use an indicator to denote if the comparison has a *p*-value of ≥0.05 = 0 or <0.05 = 1.
5. Use pairwise meta-analysis to analyze the combined data of the existing trials and the new trial. Use an indicator to denote if the comparison of C to D from the pairwise meta-analysis has a *p*-value of ≥0.05 = 0 or <0.05 = 1. This step represents for analyzing with the existing trials. Extract the Z value as *z*_2_ for future use.
6. Use an indicator to denote if (*z*_1_ > 2.16 or *z*_2_ > 2.04) = 1 else 0. This step represents for TSA.
7. Repeat steps 1–6 for 100,000 times.
8. Calculate the proportion of the indicator equal to 1. The proportion of 1’s in step 4, 5 and 6 estimates the type I error risk for the new trial, pairwise meta-analysis, and TSA separately. This is referred to as Scenario 1.
9. To simulate a scenario that the new trial will be conducted when the *p*-value in step 2 is between 0.05 and 0.1, go through steps 1–8 but revise step 3, i.e., change the threshold for *p*-value to be between 0.05 and 0.1. This is referred to as Scenario 2.
10. To simulate a scenario that the conduct of a new trial does not depend on the result of the comparison in the existing trial, go through steps 1–8 but omit step 3, i.e., the decision to conduct the trial is independent of a “promising” comparison. This is referred as Scenario 3.

## 3 Results

The results of the Simulation Study 1 are shown in Table 2. The first eight rows present scenario 1 and 2, where the decision to conduct of the new study is determined by the *p*-value of the hypothesis test from the NMA. Recall that in scenario 1, the new study was only conducted when the *p*-value of the indirect comparison is <0.1 . In scenario 2, the new study was only conducted when the *p*-value of the indirect comparison is between 0.05 and 0.1. In the simulation, a *p*-value of 0.05 is used when determining there is significant difference between two treatments so the type I error should be around 5% theoretically. The type I error risk associated with analyzing the trial without leveraging the existing network is as expected, i.e. 5%. However, the type I error risk is elevated when the new trial is analyzed with the existing network. In scenario 1, the magnitude of the elevation is the largest when the new trial is analyzed with sequential analysis. For example, when the total sample size of the new trial is 100, the type I error risk is 31.5% when analyzing with existing network while it is 39.7% under sequential analysis. In scenario 2, the magnitude of the elevation in sequential analysis is less than that in analyzing with existing network. For example, when the total sample size of the new trial is 50, the type I error risk is 20.3% when analyzing with existing network while it is 15.0% under sequential analysis. It is also notable that the type I error risk in scenario 1 is always larger than scenario 2 under the same setting of total same sample size when the new study is analyzed with the existing network or sequential analysis. This is because *p*-values are smaller in scenario 1 which leads to more false rejection when new trial data is analyzed together with existing network. The last four rows in Table 2 indicate the results in scenario 3, which is the new study will be conducted regardless of the *p*-value of the hypothesis. When the *p*-value of the indirect comparison was not used to inform the decision to conduct the trials, the type I error risk is controlled regardless of the analysis approach.

**TABLE 2:** Simulation Study 1: Type I error risk (100,000 simulation) under different scenarios of conducting new study and three analysis methods (TSA, with or w/o existing network). In the simulation, the effect size is 0 between TRIM and CEFTH. The *p*-value in the table refers to the *p*-value (*H*_0_: the effect size is 0 between TRIM and CEFTH) in the existing network.

Scenarios of conducting the new trial	Total	Type I error risk		
	Sample size	With existing network (%)	Sequential (%)	W/o existing network (%)
Scenario 1: *p*-value <0.1	50	38.5	47.3	5.2
	100	31.5	39.7	5.3
	150	27.0	33.8	5.3
	200	23.8	29.1	5.0
Scenario 2: 0.05 < *p*-value <0.1	50	20.3	15.0	5.2
	100	18.4	15.6	5.4
	150	16.4	15.0	5.5
	200	15.6	14.6	4.8
Scenario 3: regardless of *p*-value	50	4.8	5.3	5.1
	100	4.8	5.3	5.1
	150	4.9	5.2	5.3
	200	4.8	5.1	5.0

The results of Simulation Study 2 are shown in Table 3. The sample size of the new trial is fixed at 103 because the required sample size is 705 and 602 animals are in the existing network. The three rows represent for three scenarios of conducting the new trial. In first two rows, i.e., the decision to conduct of the new study is determined by the *p*-value of the hypothesis test from the pairwise meta-analysis, the type I error risk is well-controlled when analyzing without existing study while it raises when analyzing with existing study or TSA. Specifically, in scenario 1, the type I error is the largest when analyzing with existing study. In scenario 2, the type I error is the largest when using TSA. The last row shows the result of scenario 3, which is the new trial will be conducted regardless of the *p*-value of the hypothesis. As expected, the type I error risk is controlled regardless of the analysis approach.

**TABLE 3:** Simulation Study 2: Type I error risk (100,000 simulation) under different scenarios of conducting new study with a fixed sample size of 103 and three analysis methods (TSA, with existing study, and w/o existing study). In the simulation, the effect size is 0 between GAMI and FLOR. The *p*-value in the table refers to the *p*-value (*H*_0_: the effect size is 0 between GAMI and FLOR) in the existing study.

Scenarios of conducting the new trial	Type I error risk		
	With existing study	TSA	W/o existing study
Scenario 1: *p*-value <0.1	43.5%	45.7%	4.9%
Scenario 2: 0.05 < *p*-value <0.1	22.3%	16.3%	4.9%
Scenario 3: regardless of *p*-value	5.0%	4.9%	4.9%

## 4 Discussion

Combining the results of a trial with an existing network leads to increased power for estimating the relative effect (Sutton et al., 2007; Roloff et al., 2013; Nikolakopoulou et al., 2014). However, if a rationale for a trial is biased by knowledge of the result of a prior comparison, then the type 1 error risk will be inflated. For some research communities, this finding is not surprising, however for other communities it is unexpected. Indeed, the impact of “informed” approaches to leveraging information are most commonly discussed in terms of Bayesian analyses because the idea of an explicit prior is clearer in Bayesian approaches to data analysis. For example, Psioda and Ibrahim (2019) stated that all existing information must be disregarded in the analysis to control the type I error risk in a Bayesian clinical trial design. However, few clinicians use Bayesian approaches for trials so the concept of inflated type I error is unlikely to be at the forefront of considerations.

In the frequentist analysis scenario which we present, the discussion on the inflation type I errors are most frequently focused on the multiple and sequential testing when updating pairwise meta-analysis (Kang, 2021) and several methods have been proposed (Pogue and Yusuf, 1997; Hu et al., 2007; Wetterslev et al., 2017) to control the overall type I error. However, the mechanism of borrowing information for the decision of conduct of a new trial has not been examined in terms of type I error.

Our simulation study documents that the type I error risk is only controlled when we leverage the existing information appropriately. When the existing information is irrelevant to the conduction of a new trial, the type I error risk is always controlled around 5% regardless of the analysis method. When the decision to conduct of a new trial depends on the existing information, the type I error risk is increased above 5%. In other words, a new trial with predefined two treatments should not utilize the existing information in the process of conduct and analysis at the same time or the type I error risk is not desirable.

As we mentioned previously, the inflation of type I error from multiple testing associated with updating pairwise meta-analysis is well-discussed and trial sequential analysis is widely accepted when researchers make conclusions using pairwise meta-analysis^1^. For example, Whitemore et al. (2019) designed a trial, motivated by the results of the existing two trials and the analysis plan included was to combine these three trials in a pairwise meta-analysis, using a TSA method. We therefore asked an additional question, is TSA able to control the type I error introduced by the dependence between the decision to conduct a new trial and the result from existing evidence. Our simulation study proved that TSA is not successful in controlling the type I error risk in our simulated settings. When the new trial is conducted if the existing evidence of that comparison is “promising” but not significant, TSA can reduce the magnitude of the inflated type I error. When the new trial is conducted if the existing evidence of that comparison is “promising,” it makes the situation worse, which is reasonable since the significance has a high chance to appear when testing the existing evidence. Further, by borrowing the idea of TSA, we applied sequential analysis in NMA. The simulation study confirmed that applying sequential analysis in NMA is not contributed to solving the type I error problem in our case, either.

To clarify, the concern here is not about combining evidence in a prior network. The bias introduced here relates to the decision to conduct a particular trial. For future trial planners, determining the treatments included in a new trial should be independent of the prior results. If it is unavoidable, researchers should not conduct network meta-analysis by combining the existing network and the new trial. Although combining trial results in (network) meta-analysis offers opportunities to increase the value of studies by enabling indirect comparisons and offering the opportunity to leverage prior evidence to increase power, the approach should not be used to help a comparison reach significance. To date, prospectively planning to combine a new trial with a network meta-analysis is probably rare because network meta-analysis are uncommon. However, as living reviews and network meta-analysis become more common, we can envision that such an approach may be tempting. Our concern is that researchers with a vested interest in a particular comparison will look at promising results and decide to conduct another trial to try to get the result to be “significant.” Our results show that such a rationale can elevate the type I error risk. In the meantime, researchers should make the motivation for the new study clear such that later meta-analysts would treat those trials carefully. Further, new methods to analyze the trials that are motivated by prior results and the independent trials together should be explored in future research since it is common to update the network meta-analysis (Elliott et al., 2017; Simmonds et al., 2017; Thomas et al., 2017; Iannizzi et al., 2022) and eventually all relevant trials will be included, making it hard to ensure that all trials included in a network were motivated by reasons, other than prior results.

## 5 Conclusion

To conclude, the decision that a new trial is performed should not depend on a promising statistically significant finding indicated by the existing network. Such an approach will result in an increased type I error risk when the new trial is analyzed with the existing network. More importantly, the issue of inflated type I error risk cannot be solved by using sequential analysis while this increase in type I error risk does not occur if the trial is not included in a meta-analysis. However, as (network) meta-analysis is an increasingly common approach to synthesising research, we can anticipate that eventually most trials can, and will, be incorporated into a meta-analysis. Therefore, even if the researchers conducting the trial have no intention of combining the result with a meta-analysis, it can be anticipated this will occur perhaps by other researchers. Therefore, it is preferred to have the decision of conducting a trial independent of the prior results, and researchers should be transparent when the design of any trial was motivated by a promising result in a meta-analysis.

## Data Availability

The original contributions presented in the study are included in the article/Supplementary Material, further inquiries can be directed to the corresponding author.

## Footnotes

1 Copenhagen Trial Unit. PUBLICATIONS INVOLVING TRIAL SEQUENTIAL ANALYSIS (TSA) (2020). https://ctu.dk/tsa/publications/ [Accessed 28 May 2022].
